# Pioglitazone Improves Fat Distribution, the Adipokine Profile and Hepatic Insulin Sensitivity in Non-Diabetic End-Stage Renal Disease Subjects on Maintenance Dialysis: A Randomized Cross-Over Pilot Study

**DOI:** 10.1371/journal.pone.0109134

**Published:** 2014-10-16

**Authors:** Anne Zanchi, Luc Tappy, Kim-Anne Lê, Murielle Bortolotti, Nicolas Theumann, Georges Halabi, Thierry Gauthier, Claudine Mathieu, Sylvie Tremblay, Pauline Coti Bertrand, Michel Burnier, Daniel Teta

**Affiliations:** 1 Service of Nephrology, Department of Medicine, Lausanne University Hospital, Centre Hospitalier Universitaire Vaudois, Lausanne, Switzerland; 2 Department of Physiology, University of Lausanne, Lausanne, Switzerland; 3 Service of Endocrinology, Diabetes and Metabolism, Department of Medicine, Lausanne University Hospital, Centre Hospitalier Universitaire Vaudois, Lausanne, Switzerland; 4 Department of Radiology, Lausanne University Hospital, Centre Hospitalier Universitaire Vaudois, Lausanne, Switzerland; Weill Cornell Medical College Qatar, Qatar

## Abstract

**Background:**

Fat redistribution, increased inflammation and insulin resistance are prevalent in non-diabetic subjects treated with maintenance dialysis. The aim of this study was to test whether pioglitazone, a powerful insulin sensitizer, alters body fat distribution and adipokine secretion in these subjects and whether it is associated with improved insulin sensitivity.

**Trial Design:**

This was a double blind cross-over study with 16 weeks of pioglitazone 45 mg vs placebo involving 12 subjects.

**Methods:**

At the end of each phase, body composition (anthropometric measurements, dual energy X-ray absorptometry (DEXA), abdominal CT), hepatic and muscle insulin sensitivity (2-step hyperinsulinemic euglycemic clamp with 2H2-glucose) were measured and fasting blood adipokines and cardiometabolic risk markers were monitored.

**Results:**

Four months treatment with pioglitazone had no effect on total body weight or total fat but decreased the visceral/sub-cutaneous adipose tissue ratio by 16% and decreased the leptin/adiponectin (L/A) ratio from 3.63×10^−3^ to 0.76×10^−3^. This was associated with a 20% increase in hepatic insulin sensitivity without changes in muscle insulin sensitivity, a 12% increase in HDL cholesterol and a 50% decrease in CRP.

**Conclusions/Limitations:**

Pioglitazone significantly changes the visceral-subcutaneous fat distribution and plasma L/A ratio in non diabetic subjects on maintenance dialysis. This was associated with improved hepatic insulin sensitivity and a reduction of cardio-metabolic risk markers. Whether these effects may improve the outcome of non diabetic end-stage renal disease subjects on maintenance dialysis still needs further evaluation.

**Trial Registration:**

ClinicalTrial.gov NCT01253928

## Introduction

Patients with ESRD on maintenance dialysis are prone to body fat redistribution with an excess of visceral adipose tissue (VAT), relative to subcutaneous adipose tissue (SAT). This pattern of fat distribution is associated with dyslipidemia [1] and inflammatory cytokine production [2], all intimately linked with insulin resistance (IR). Abdominal obesity, as well as truncal fat distribution without obesity, predict all cause and cardiovascular mortality in patients with ESRD [3].

Furthermore, adipose tissue accumulation coupled with low glomerular filtration rate may lead to the accumulation of plasma adipokines. Among adipokines, lower levels of adiponectin have been associated with the development of IR, whereas leptin is directly correlated with body fat and nutritional intake [4], [5]. In ESRD, leptin accumulates to a much larger extent than adiponectin, thus producing an elevated plasma leptin to adiponectin (L/A) ratio [6]. Plasma L/A ratio is identified as the best correlate of IR measured by the hyperinsulinemic euglycemic clamp (HEGC) in African American patients with ESRD on maintenance hemodialysis [7]. The L/A plasma ratio is also considered as an atherogenic index in the general population [8], and in patients with type 2 diabetes (T2DM) [9]. Thus, a high L/A ratio in ESRD may play a pivotal role in the pathogenesis of both IR and cardiovascular complications in ESRD.

Glitazones are effective insulin sensitizers available in clinical practice and their use is associated with significant and favourable changes in body fat distribution and adipokine profile in subjects with T2DM [10]. Furthermore, glitazones have also been shown to improve glucose metabolism in diabetic and non diabetic ESRD subjects [11]–[13]. Whether these effects are related to glitazone-induced changes in body fat distribution or to changes in blood L/A ratio is not known.

The aim of this study was to evaluate whether pioglitazone alters body fat distribution and the L/A ratio in non diabetic subjects on maintenance dialysis, and whether this was associated with alterations in insulin sensitivity. Pioglitazone significantly changed the visceral-subcutaneous fat distribution and plasma L/A while improving hepatic insulin sensitivity.

## Subjects and Methods

**Figure 1:**
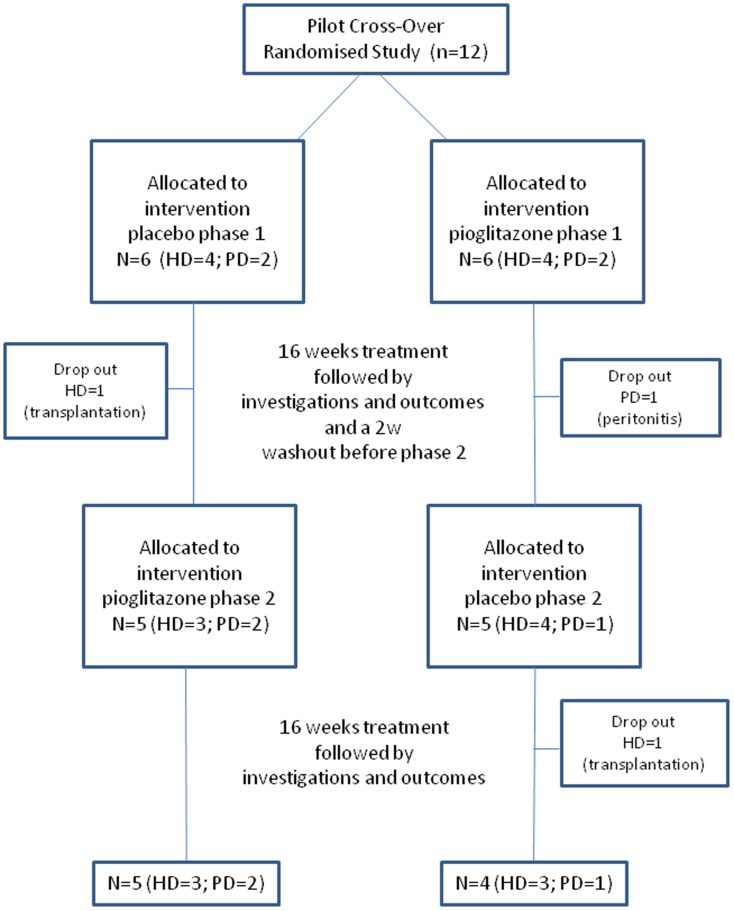
Flow chart of the double blinded cross-over study.

The study design was double blind randomized cross-over with 2 phases (Figure 1) and conducted at the University Hospital of Lausanne, Switzerland (CHUV). In 2007, it received the approvals from the local Ethics Committee (Human Research Ethics Committee, Lausanne) and from Swissmedic (Swiss Agency for Therapeutic Products) according to the principles expressed in the declaration of Helsinki. At the time of the submission, the local ethics committee did not require the registration in ClinicalTrial.gov. For this reason, the trial was registered only while it was ongoing in 2010 under ClinicalTrial.gov NCT01253928. The protocol for this trial and supporting CONSORT checklist are available as supporting information; see Checklist S1 and Protocol S1 and S2. This manuscript focuses exclusively on data of body composition, adipokines, glucose metabolism and insulin resistance. Non diabetic patients with ESRD treated for more than 3 months in the dialysis centre of the CHUV and in related centers of the Canton de Vaud, were recruited after informed and written consent. Recruitment took place between 2008–2010 and followed up until 2011. Diabetic patients were excluded to prevent the confounding factor of insulin treatment on IR assessment. Other exclusion criteria were: estimated survival less than 6 months, active infection, hospitalization within 1 month before the study, congestive heart failure NYHA class III–IV and abnormal liver function tests. The sample size chosen for this pilot study was consistent with other studies having used this technique in this population [7], [14], [15]. For this study, twelve subjects were included, of which 8 were on hemodialysis (HD; age: 59.6±4.4 years) and 4 on peritoneal dialysis (PD, age: 43.5±3.6 years). The average body mass index (BMI) was of 27.2±0.74 kg/m^2^ (range: 24.0–30.5 kg/m^2^).

Each subject underwent the 2 treatment phases lasting 16 weeks each, separated by a wash-out period of at least 2 weeks (Figure 1). The sequence of treatment was randomized following allocation to random numbers with the objective to have an equal number of individuals starting with pioglitazone or placebo. The medical staff providing the random allocation sequence differed from those blinded for the treatment allocation involved in the recrutement, and interventions. For those starting with the pioglitazone phase, the following 18 week period without treatment (washout and placebo phase) was considered sufficient to avoid a significant carry-over effect of the pioglitazone phase. A similar design was used recently in a study examining the effects of pioglitazone on insulin sensitivity and sub-cutaneous adipose cell morphology [16]. The primary outcome was changes in visceral/subcutaneous abdominal fat distribution. Secondary outcomes were changes in the L/A ratio, in hepatic and muscle insulin sensitivity and in biochemical cardiovascular risk markers.

At the end of each treatment, subjects underwent anthropometric [17] and DEXA measurements and an abdominal CT scan at the level of L4–L5 for the measurement of abdominal fat distribution. For HD patients, these measurements were performed immediately after an HD session, whereas for PD patients, they were performed after the morning PD effluent drainage.

During the whole study, subjects were followed by their nephrologist. For HD patients, dry body weight and ultrafiltration rates (UF) were recorded at each HD session. A clinical examination was performed once a week with special focus on the presence of lower extremity edema and on other signs of fluid overload. For PD patients, dry weight was recorded each morning at bedside and daily UF rates were registered. Patients were instructed to report each PD exchange and solutions on a flow sheet. Patients were seen every 4 weeks by their nephrologist who performed a clinical examination with special focus on the presence of lower extremity edema and on other signs of fluid overload. In addition, a weekly phone call was made to the subject to check for potential side-effects and/or signs of fluid overload.

### Metabolic investigations

Subjects reported at 0700 am to the metabolic unit, after a 10 h overnight fast. HD patients were investigated in the metabolic unit the day following an HD session. PD patients were investigated after their first PD exchange in the morning. On arrival, subjects were asked to empty their bladder and body composition was estimated from subcutaneous skin-fold measurements at the biceps, triceps, subscapular and suprailiac sites [17]. An indwelling catheter was inserted into an antecubital vein of the right wrist for repeated blood sampling. A second indwelling catheter was inserted into an antecubital vein of the other arm for glucose, insulin and tracer infusions. If the patient had an arteriovenous fistula placed on the upper limb, this was accessed using a 16 gauge fistula needle for infusions of glucose, insulin and tracers, and the blood sampling catheter was placed on the other side. Blood was collected at baseline for the measurement of plasma concentrations of glucose, insulin, total, HDL and LDL cholesterol, triglycerides, CRP, IL-6, leptin and adiponectin. Monthly measurements of serum albumin (immunonephelemetry), creatinine, and normalized protein catabolic rate (nPCR) were provided by the dialysis centers in order to complete the assessment of nutritional status and to estimate daily protein intake.

#### Methods for the 2-step hyperinsulinemic euglycemic clamp

Liver and muscle IS were measured for 3 h after the initial 2 h tracer infusion. A 2-step hyperinsulinemic euglycemic clamp (0.03 mU · kg^−1^ · min^−1^ and 1 mU · kg^−1^ · min^−1^, 90 min each), was performed and aimed to achieve a glycemia of 5.5 mmol/l, regardless of the patients' fasting blood glucose. Blood samples were collected every 5 min during the clamp to monitor plasma glucose concentration and at 30 min intervals for the analysis of tracers and insulin. Glucose metabolism was assessed during the 2-step clamp with the continuous infusion of 6,6-[^2^H_2_]glucose infusion (Cambridge Isotope Laboratories, Inc. Andover, MA; bolus: 2 mg/kg; continuous 20 µg · kg^−1^ · min^−1^) and the plasma measurements of 6,6-[^2^H_2_]glucose. Substrate oxidation was measured by indirect calorimetry (ventilated canopy) from 0800 to 1300 [18] by using the equations of Livesey and Elia [19].

Glucose appearance rates were calculated at baseline and during moderate and high insulinemia from plasma 6,6 ^2^H_2_ glucose Steele's equations for steady state conditions [20]. Endogenous glucose production (EGP) was identical with glucose appearance rate in fasting conditions. During hyperinsulinemia, EGP was computed as (glucose appearance rate) - (glucose infusion rate). Since all measurements were done under steady state conditions, total glucose disposal rate was considered as equal to glucose appearance.

### Analytical determinations

Plasma was immediately separated from blood by centrifugation at 4°C for 10 min at 3600 rpm and stored at −20°C. Commercial radioimmunoassy kits were used to measure plasma insulin, leptin, and adiponectin (LINCO Research, St Charles, MO). During the clamp, plasma glucose concentrations were measured by the glucose oxidase method with a Beckman glucose analyzer II (Beckman Instruments, Fullerton, CA). Plasma 6,6-[^2^H_2_]glucose isotopic enrichment was measured by gas chromatography-mass spectrometry (Hewlett-Packard Instruments), as previously described [21]. Breath ^13^CO_2_ isotopic enrichment was determined by Isotope Ratio Mass Spectrometry (IR/MS) on a Tracermass C/N (SerCon Ltd, Crewe, Cheshire, UK). The homeostatic model assessment of insulin resistance index (HOMA-IR) and the HOMA corrected by adiponectin index (HOMA-AD) were calculated according to published formulas [7].

## Statistical Analysis

Because dialysis patients are heterogeneous, we chose to perform a cross-over design instead of a case-control study. This design increases the power of the study as each individual examined serves as their own control. Taking into account the within patient standard deviation and expected differences between treatments for body fat distribution and for adiponectin plasma levels based on previous studies using the same dosage of pioglitazone and same duration of study [10], [22], a sample size of 12 individuals was chosen with a projected drop-out rate of 10–20% to reach the respectively 10 and 7 patients required to detect a treatment difference for body fat distribution (primary end point) and adiponectin plasma levels. At the end of each period (pioglitazone and placebo), absolute values of parameters were paired and compared as in previous publications using the same design [16], [23], [24]. Data were expressed as mean ± SEM. The statistical differences between the 2 periods of treatment (placebo and pioglitazone) were analysed by the paired Student's t test using the Minitab software. A level of p<0.05 was considered statistically significant. The analysis of variance (ANOVA) for paired comparisons was performed to examine an overall effect of treatment during the clamp studies and whether the sequence of treatments influenced the response to treatment (carry over effect). The residual method was used when examining the effects of pioglitazone while adjusting for co-variables. Briefly, this procedure uses a linear regression to assess the effect of confounders on the dependent variable, and then computes the residual (difference between observed and predicted value). The residuals were then compared using a standard paired t-test.

## Results

Nine subjects completed both phases of the study (**Table 1**). Two HD subjects dropped out of the study due to kidney transplantation. One PD subject dropped out because of peritonitis and subsequently switched to HD.

**Table 1:** Characteristics of subjects enrolled and having completed both phases.

Subject	Age	Sex	Ethnicity	Cause of ESRD	Duration of dialysis (months)	Dialysis modality
1	68	M	W	Ischemic nephropathy and renal cholesterol emboli	52	HD
2	71	M	W	IgA nephropathy	61	HD
3	53	M	A	Obstructive uro-nephropathy	17	HD
4	79	M	W	Hypertensive nephroangiosclerosis	49	HD
5	57	M	W	Post-streptococcal Glomerulo-nephritis	124	HD
6	40	M	W	Polycystic kidney disease	42	HD
7	39	F	W	Reflex uro-nephropathy	35	PD
8	42	F	W	Membrano-proliferative glomerulonephritis	37	PD
9	54	M	W	Hypertensive nephroangiosclerosis	40	PD

M: male, F: female, W:White, A: African, HD: hemodialysis, PD: peritoneal dialysis.

### Body Composition

The body composition of subjects, at termination of each phase, is presented in **Table 2**. Pioglitazone had no effect on total body weight, but significantly decreased abdominal VAT and the VAT/SAT ratio (mean ± SEM; n = 9, placebo: 0.69±0.09, pioglitazone: 0.58±0.09, p = 0.002). After stratification for dialysis modality, in HD patients VAT/SAT ratio was of (mean ± SEM; n = 6) placebo: 0.84±0.06, pioglitazone: 0.72±0.07, p = 0.01. In PD patients VAT/SAT ratio was of (mean ± SEM; n = 3) placebo: 0.37±0.1, pioglitazone: 0.29±0.04, p = 0.2. These changes were similar in both dialysis modalities although it did not reach a significant level in PD because of the small number of observations. Subscapular subcutaneous fat deposition significantly increased. In contrast, there was no effect on lean mass, and on bone mineral density (BMD).

**Table 2:** Body composition measurements at termination of placebo and pioglitazone phases.

	Placebo	Pioglitazone	*Diff (95%CI)*	*P*
Weight (kg)	78.4±2.2	78.1±2.4	0.35 (−0.7;1.4)	
Delta weight from start to end of phase	0.51±0.48	0.69±0.35	−0.18 (−1.7;1.3)	
BMI (kg/m^2^)	27±0.8	26.7±0.8	0.2 (−0.3;0.8)	
Waist circumference (cm)	103.6±2.9	103.6±3.3	0 (−1.2;1.2)	
Subscapular skinfold (cm)	16±1.2	19.1±1.5	−3.1 (−6.0;−0.2)	0.04
Biceps skinfold (cm)	9.3±1.6	8.0±1.6	1.39 (−1.2;4.0)	
Triceps skinfold (cm)	20.6±2.7	19.1±2.5	1.6 (−1.0;4.1)	
Brachial muscular circumference (mm)	272.2±4.6	271±4.8	1.3 (−4.6;7.1)	
**DEXA measurements**				
DEXA fat (kg)	26.1±2.1	25.7±2.2	0.4 (−1.1;1.8)	
DEXA fat (%)	33.3±2.5	33.1±2.8	0.2 (−1.5;1.9)	
DEXA leg fat (kg)	7.8±0.9	8.1±1.0	−0.2 (−0.7;0.3)	
DEXA arm fat (kg)	2.9±0.4	3.1±0.4	−0.17 (−0.5;0.12)	
Lean mass (kg)	49.9±2.3	49.8±2.4	0.2(−1.0;1.4)	
Lean mass: leg (kg)	15.4±0.9	15.1±0.9	0.3 (−0.2;0.7)	
Lean mass: arm (kg)	4.7±0.6	5.3±0.4	−0.7 (−1.9;0.6)	
BMD (g/cm2)	1.09±0.03	1.09±0.03	0 (−0.02;0.01)	
BMD spine (g/cm2)	0.92±0.03	0.94±0.05	−0.01 (−0.10;0.07)	
BMD pelvis (g/cm2)	1.08±0.06	1.08±0.05	0 (−0.03;0.03)	
BMD arm (g/cm2)	1.44±0.16	1.63±0.05	−0.20 (−0.65;0.26)	
**Abdominal CT scan measurements**				
VAT (cm2)	18.3±2.8	15.9±2.8	2.3 (0.9;3.8)	0.005
SAT (cm2)	27.6±2.4	28.5±2.7	−0.9 (−2.3;0.6)	
VAT/SAT ratio	0.69±0.09	0.58±0.09	0.11 (0.05;0.12)	0.002

VAT: visceral adipose tissue area; SAT: subcutaneous adipose tissue area, DEXA: dual energy X-ray absorptiometry, BMD: bone mineral density.

### Fasting blood chemistry

**Figure 2:**
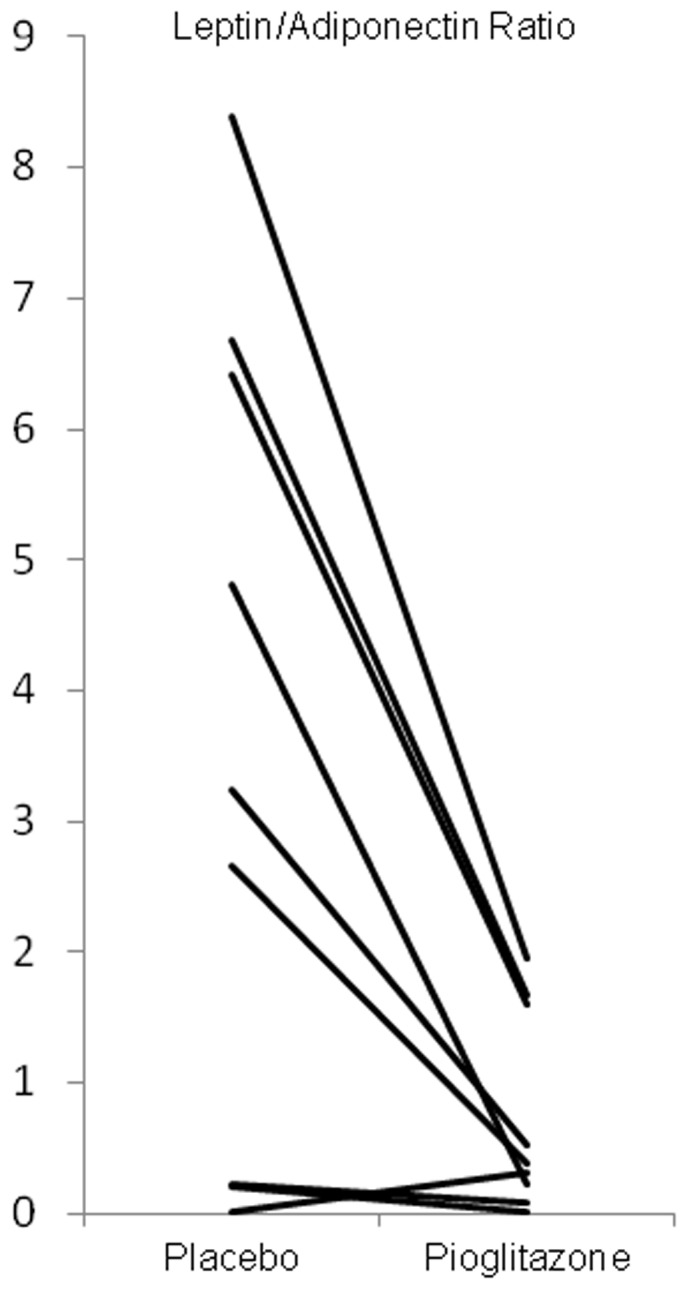
Individual changes in the leptin/adiponectin ratio.

**Table 3** summarizes the biochemical analysis at termination of each phase. Plasma albumin, creatinine and nPCR were not affected by pioglitazone. HDL cholesterol increased significantly with pioglitazone. There was a trend toward a decrease in CRP and IL-6 plasma levels in accordance with a study performed in hemodialysis patients [25], [26]. Under pioglitazone, plasma leptin concentrations significantly decreased, whereas plasma adiponectin significantly increased, thus resulting in a dramatically suppressed L/A ratio (**Figure 2**; mean ± SEM; n = 9, placebo: 3.63±1.04, pioglitazone: 0.76±0.25, p = 0.008). These effects remained significant when adjusting the L/A ratio to visceral fat, total glucose disposal rate (GDR), or hepatic glucose output. After stratification for dialysis modality, in HD patients L/A ratio was of (mean ± SEM; n = 6) placebo: 3.85±1.37, pioglitazone: 0.79±0.32, p = 0.039. In PD patients L/A ratio was of (mean ± SEM; n = 3) placebo: 3.18±1.88, pioglitazone: 0.69±0.5, p = 0.2. These changes were similar in both dialysis modalities although it did not reach a significant level in PD patients because of the small number of observations.

**Table 3:** Biochemistry values at termination of placebo and pioglitazone phases.

	Placebo	Pioglitazone	*Diff (95%CI)*	*P*
Cholesterol (mmol/l)	3.99±0.21	3.99±0.21	−0.03 (−0.43;0.37)	
HDLcholesterol (mmol/l)	0.65±0.06	0.73±0.04	−0.09 (−0.17;0)	0.047
Triglyceride (mmol/l)	1.47±0.17	1.67±0.18	−0.19(−0.62;0.23)	
LDL cholesterol (mmol/l)	2.81±0.25	2.53±0.29	0.28 (−0.1;0–65)	
Albumine (g/l)	40.0±2.2	39.1±1.9	1.0 (−3.7;5.6)	
nPCR (g/kg/day)	0.99±0.10	1.03±0.12	−0.04 (−0.16;0.08)	
Creatinine (µmol/l)	800±64	819±63	−19 (−92;54)	
CRP (mg/l)	7.88±2.56	3.96±1.44	3.92 (−0.44;8.28)	0.07
IL-6 (pg/ml)	6.11±4.4	3.6±2.5	2.52 (−2.34;7.39)	
Glucose (mmol/l)	5.1±0.2	4.8±0.1	0.3 (0.03;0.6)	0.04
Insulin (µU/ml)	9.8±1.3	8.1±0.8	1.8 (−0.2;3.8)	
HOMA-IR	2.3±0.3	1.75±0.2	0.5 (0;1)	0.05
HOMA-AD	120±35	46±14	74.7 (−18.7;168.1)	
Leptin (ng/ml)	54.0±16.5	36.7±15.9	17.3 (0.87;33.7)	0.04
Adiponectin (µg/ml)	22.6±5.1	49.1±9.0	−26.5 (−51.8;1.2)	0.04
Leptin/Adiponectin ratio	3.63×10^−3^±1.04×10^−3^	0.76×10^−3^±0.25×10^−3^	2.87 (1.0;4.8)	0.008

nPCR: normalized protein catabolic rate, HOMA-IR: Homeostatic model assessment of insulin resistance, HOMA-AD: HOMA corrected by adiponectin, NEFA: non-esterified fatty acids.

### Glucose homeostasis (Table 3, Figure 3)

**Figure 3:**
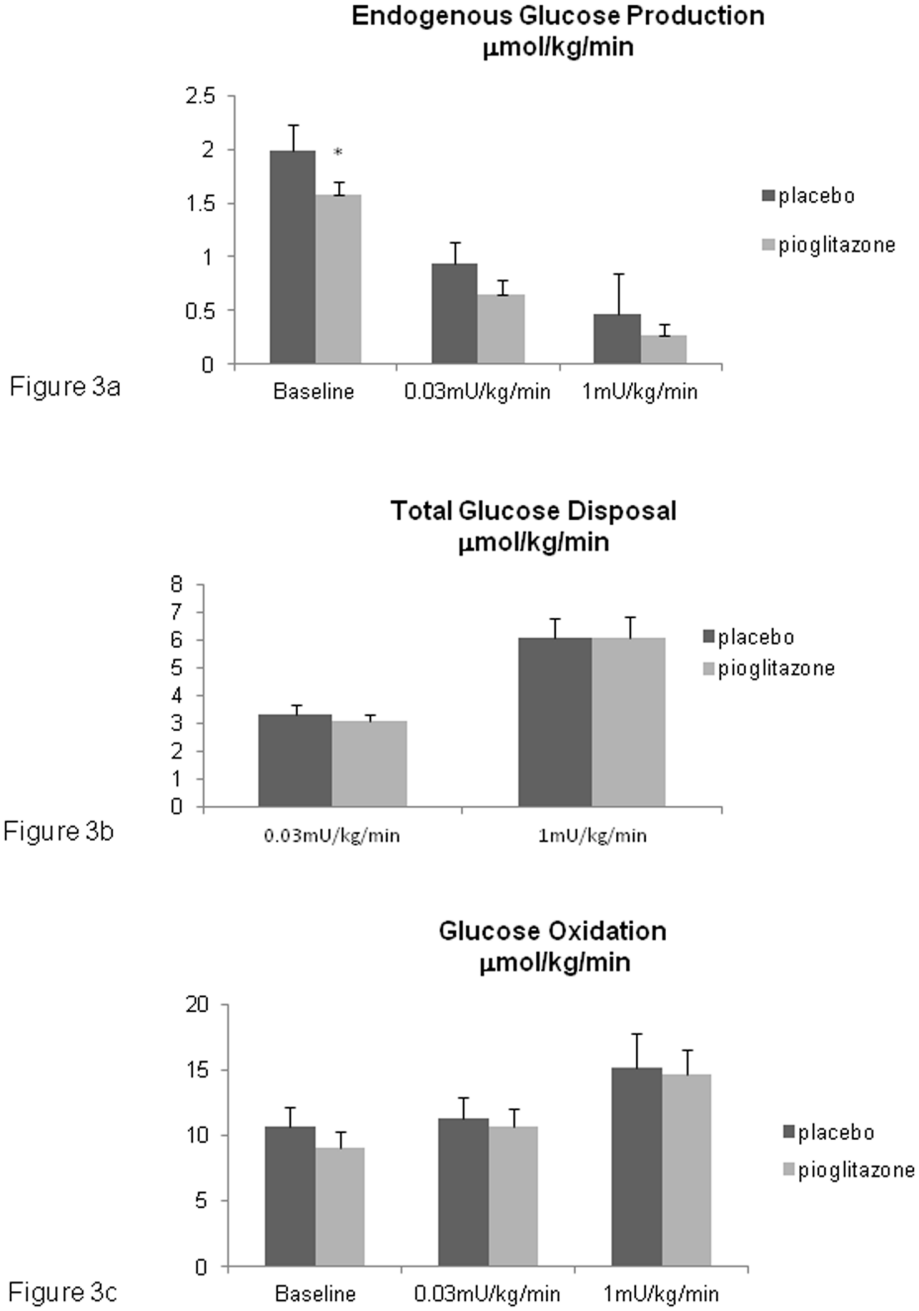
Glucose metabolism during a 2-step HEGC, values at baseline and during insulin infusion rates of 0.03 mU/kg/min and 1 mU/kg/min. * p<0.05.

Pioglitazone significantly decreased fasting blood glucose and HOMA-IR was lower with pioglitazone than with placebo (resp. 1.75±0.2 vs 2.3±0.3; p = 0.05). Hepatic glucose output (endogenous glucose production) was significantly decreased (−20.6%, Figure 3a) with pioglitazone at baseline, demonstrating an improvement in hepatic IS with pioglitazone. This difference was maintained, although not significantly, at low and high dose insulin infusion rates. There was a trend towards an increase in the hepatic insulin sensitivity index (HISI, +56.8%, p = 0.06). As at baseline, GDR is equivalent to hepatic glucose production, the effects of insulin on GDR are only presented during the insulin infusion steps (Figure 3b). At low dose and high dose insulin infusion rates, pioglitazone did not significantly improve GDR. As expected, glucose oxidation was increased with insulin infusion (Figure 3c). Pioglitazone did not influence glucose oxidation at baseline or during insulin infusions.

In order to evaluate a possible carry-over effect, we compared the values of VAT and L/A ratio among subjects starting with the placebo phase (n = 5) versus subjects starting with the pioglitazone phase (n = 4). The difference of pioglitazone-induced changes in VAT for placebo vs pioglitazone starters was respectively of (mean±SEM) −2.5 cm^2^±0.8 (−13.7%±3.6; p = 0.033)) and −2.1 cm^2^±0.8 (−15.7% cm^2^±6.6; p = 0.147) suggesting that there may be a carry-over effect of pioglitazone on VAT with a consequent decreased difference in pioglitazone starters. The differences of pioglitazone-induced changes in L/A ratio for placebo or pioglitazone starters was respectively of (mean±SEM) −2.03±0.87; p = 0.081 and −3.93±1.5; p = 0.075. Thus, for these parameters, having started with pioglitazone or placebo did not have a significant impact on the pioglitazone-induced changes compared to placebo. The numbers are indeed low and a much larger study would be required to explore a possible carry-over effect.

## Safety

No significant side effects were observed during pioglitazone therapy. Three patients were hospitalized for reasons unrelated with the use of pioglitazone (see above) and were dropped out. None of the patients developed signs of fluid retention and interdialytic weight gains and UF volumes were comparable between 2 phases.

## Discussion

Insulin resistance is common in subjects with chronic kidney disease and ESRD [27], [28] and is associated with worse cardio-vascular outcomes [29]. Mechanisms involved in the “uremic IR state” are multiple [28]. Among them, increase in visceral fat, change in adipokines, chronic inflammation, are modified by the IS properties of pioglitazone in patients with normal renal function [22]. Yet, these effects have not been studied in ESRD patients. We therefore assessed whether pioglitazone alters the body fat distribution in non diabetic ESRD subjects and whether it is associated with an improvement in insulin sensitivity and cardiometabolic risk factors.

We show that the short term use of pioglitazone is safe in ESRD patients treated by maintenance dialysis and leads to a change in body fat distribution. Although total body fat did not change, there was a significant decrease in the VAT abdominal area, and in the VAT/SAT ratio, indicating a redistribution of visceral fat toward the subcutaneous area as demonstrated by the significant increase in fat at the subscapular site. These changes in fat distribution were associated with significant metabolic changes. The adipokine profile changed favourably with a strong suppression of the high L/A ratio to levels close to those found in the general population [30]. HDL-cholesterol increased and there was a decrease in inflammation. Hepatic insulin sensitivity significantly improved, as documented by a lower fasting hepatic glucose production and an increase in the hepatic insulin sensitivity index. This is consistent with visceral fat playing a key role in the development of hepatic insulin resistance and inflammation.

This study is the first to examine the effects of glitazones on insulin sensitivity in ESRD subjects, using the HEGC technique. HEGC is certainly the gold standard but is a complicated procedure and in general, only surrogate indicators of IR have been studied in previous reports. In non ESRD T2DM subjects, pioglitazone primarily suppresses endogenous glucose production (EGP) and some studies show an enhancement of insulin-induced GDR [22], [31], [32]. Our study population differs from these studies as none were T2DM, and all were on HD or PD and presented with only slight or moderate IR [7], [27]. Our results show that the uremic state did not interfere with the favorable effects of pioglitazone on body fat redistribution, adiponectin levels, EGP and on inflammation. However, there were no alterations of insulin-mediated glucose disposal. Since this is known to be essentially mediated at high insulinemia, it indicates either no effect of pioglitazone on muscle sensitivity in these patients, or more likely, these non diabetic individuals have a smaller degree of muscle insulin resistance compared to hepatic insulin resistance.

Pioglitazone lead to a suppression of the L/A ratio close to levels found in the general population. The respective contribution from subcutaneous and visceral fat to plasma leptin and adiponectin levels is difficult to assess because many factors other than fat mass may determine plasma leptin and adiponectin, especially in dialysis patients. Furthermore a direct effect of pioglitazone on the gene expression of adipokines has been demonstrated [33]–[35] and may contribute to the observed findings in this study. Although the relationship between adiponectin levels and visceral fat mass is less clear in patients with chronic kidney disease than in the general population, adiponectin has been inversely associated with visceral fat in a cohort of patients with ESRD [36]. The pioglitazone-induced increase in adiponectin found in this study confirms previous findings [26], [37], [38] and could be related to the decrease in visceral fat. This effect indeed can be considered as positive as high stable adiponectin levels were found to be associated with more favorable cardiovascular outcomes in patients with ESRD [39]. The decrease in leptin with pioglitazone is a new finding. Glitazones suppress leptin rodent gene expression [33], [34] and decrease by 40% leptin production from human adipocyte cultures [40], but have no effect on plasma levels of leptin [22]. However, the absence of an increase in plasma leptin in those studies despite fat gain may mask a glitazone-induced decrease in leptin production per unit of fat mass. In the current study, the absence of gain in total fat mass with pioglitazone may have facilitated the observed decrease in plasma leptin due to downregulation of leptin gene expression. Indeed, the decrease in plasma leptin is interesting, since leptin may be considered as a uremic toxin in ESRD [41].

While stratifying for dialysis modality, similar changes for body composition and adipokines were observed in HD and PD patients. These changes were significant for HD patients but not for PD patients because of the small number of observations. These results show that the effects of pioglitazone are independent from dialysis modality.

This study has several strengths. First, we used the gold standard methods to measure abdominal body fat distribution and insulin sensitivity. Second, we used a double-blinded randomized design, which enabled to compare the effects of pioglitazone versus placebo in the same patient. The limitations of the study include the small number of patients studied due to the complexity and laborious nature of the procedure. However the sample size is consistent with other studies having used this technique in this population [7], [14], [15]. In addition, a possible carry-over effect cannot be excluded. If all subjects had started with the placebo phase, the effects of pioglitazone may have been even greater than observed with this cross-over design.

In conclusion, 16 week treatment of pioglitazone in non diabetic ESRD subjects on maintenance dialysis is well tolerated, reduces visceral fat and improves the adipokine profile with a decrease in hepatic insulin resistance. Whether these effects may improve the outcome of non diabetic ESRD patients still needs further evaluation.

## Supporting Information

Checklist S1 **CONSORT Checklist.**
(DOCX)

Protocol S1 **Trial Protocol (original).**
(DOC)

Protocol S2 **Trial Protocol (translation).**
(DOCX)
