# Supplementary material for: Pioglitazone Improves Fat Distribution, the Adipokine Profile and Hepatic Insulin Sensitivity in Non-Diabetic End-Stage Renal Disease Subjects on Maintenance Dialysis: A Randomized Cross-Over Pilot Study
Source: PLoS One. 2014 Oct 16;9(10):e109134. doi: 10.1371/journal.pone.0109134 (PMC4199598; doi:10.1371/journal.pone.0109134)
Supplement: Protocol S1 — Trial Protocol (original). (DOC) [file pone.0109134.s002.doc]

|  | **CENTRE HOSPITALIER UNIVERSITAIRE VAUDOIS** |
| --- | --- |

| **Département de médecine**  **Division de néphrologie** |  |
| --- | --- |

# Titre de l’étude : Effets des glitazones sur la composition corporelle chez les patients traités par dialyse

**Version 3 amendée le 11.01.07**

**Investigateur responsable :** Prof. Michel Burnier

**Collaborateurs :** Dr PD A Zanchi, MD, Service de Néphrologie

Dr Teta MD-PhD, Service de Néphrologie

Prof L Tappy, MD, Département de physiologie

Dr PD-MER N Theumann, MD, Service de radiologie

Dr G Halabi, MD, Service de Néphrologie

Dr T Gauthier MD, Service de Néphrologie

Dr M Maillard, PhD, Service de Néphrologie

Prof M Roulet, MD, Unité de Nutrition clinique

**Lieu de l’étude :** Service de Néphrologie, CHUV

**Introduction.**

Les patients qui présentent une insuffisance rénale terminale, traités par dialyse sont caractérisés par une mortalité cardiovasculaire élevée, une artériosclérose accélérée, une hypertension artérielle prévalente, une redistribution du tissus adipeux de type centrale, une résistance à l’insuline, une dénutrition protéino-énergétique fréquente et un état micro-inflammatoire.

Les glitazones sont des antidiabétiques oraux qui diminuent la résistance à l’insuline. Ces médicaments sont des agonistes des récepteurs PPAR-γ dont l’expression prédomine au niveau du tissu adipeux. Bien que prescrits exclusivement aux diabétiques de type 2, les glitazones ont des effets pléiotropiques qui vont bien au delà du contrôle de la glycémie. En particulier, les glitazones ont une activité antiathérogène, diminuent la pression artérielle et diminuent l’inflammation. Ces propriétés en font des médicaments potentiellement bénéfiques pour les patients dialysés.

Les glitazones favorisent une redistribution du tissus adipeux vers le tissus sous-cutané. En stimulant la différentiation de pré-adipocytes en adipoctes “métaboliquement plus favorables”, les glitazones améliorent le profil métabolique et le contrôle de la pression artérielle. La prise pondérale est en moyenne de 3kg[1]. Certaines études montrent que parallèlement à l’augmentation du tissu adipeux sous-cutané, le tissu adipeux viscéral diminue[2, 3]. Comme les glitazones n’ont pas d’effet mesurable sur la dépense énergétique basale ou post-prandiale[2], la prise pondérale pourrait être liée à une augmentation de l’apport calorique (estimé dans cette étude à environ 175kcal/j), bien que les questionnaires sur la faim et la satiété n’ont pas montré de différences entre le groupe placebo et traité par glitazones. Nos expériences animales montrent par contre clairement que les glitazones augmentent l’apport calorique (qui peut être mesuré avec précision) dans un modèle d’insulino-résistance.

La technique de dialyse a un impact sur la composition corporelle. En particulier, les patients traités par dialyse péritonéale (DP) accumulent en général davantage de tissus adipeux que les patient traités par hémodialyse (HD). Par conséquent, il est crucial d’investiguer si l’impact des glitazones sur la composition corporelle est différent dans ces deux types de traitement.

Deux études cliniques préliminaires ont déjà démontrés que les glitazones peuvent être administrés avec sécurité chez des patients traités par HD et DP[4, 5]. Les études pharmacocinétiques montrent que la dose ne doit pas être ajustée et qu’il n’y a pas d’accumulation de la pioglitazone, de la rosiglitazone ou de leurs métabolites[6, 7, 8, 9]. Les effets secondaires les plus sérieux, à savoir l’œdème aigu du poumon et la rétention hydrosodée, ne semblent pas plus fréquents que ceux attendus dans cette population[4, 5].

## But.

Le but de l’étude est d’investiguer l’effet des glitazones sur la composition corporelle, la pression artérielle, l’appétit, la résistance à l’insuline, les adipokines plasmatiques et la CRP sensible, chez des patients traités par HD et DP.

Nous nous attendons à ce que ce traitement diminue la pression artérielle, diminue les paramètres inflammatoires et favorise une redistribution adipeuse vers le tissu adipeux sous-cutané tout en améliorant l’insulino-résistance et l’état de nutrition du patient. Dans ce cas, et si la tolérance médicamenteuse est bonne, ce traitement pourrait diminuer les risques cardiovasculaires et de dénutrition à long terme dans cette population à risque.

**Design de l’étude.**

Etude mono-centrique, randomisée, en double aveugle, en cross-over, glitazone versus placebo. Détail d’une phase de 16 semaines.

## Critères d’inclusion.

1. Patients traités par HD ou DP depuis au moins 3 mois. Les patients seront recrutés au centre de dialyse de Lausanne.

2. Pas d’hospitalisation, pas de complication infectieuse dans les 3 mois qui précèdent l’inclusion.

4. CRP < 20.

5. Signature du consentement

## Critères d’exclusion.

1. Autre étude protocolée en cours.

2. Patients déjà sous glitazone ou insuline.

3. Survie estimée à < 6 mois.

4. Insuffisance cardiaque stade III et IV selon la NYHA.

5. ASAT, ALAT > 2.5 fois la norme supérieure et/ou maladie hépatique active.

6. Contre-indications de la spectro-IRM :

Porteur de pace-maker

Antécédents de chirurgie orthopédique ou de chirurgie ophtalmique

Présence de corps étrangers métallique dans l’organisme (clip vasculaire, etc.).

7. Femmes pré-ménopausées, sans contraception orale.

8. Test de grossesse positif (fait chez toute femme pré-ménopausée sous contraception orale).

**Protocole.**

Les patients seront randomisés en 2 groupes. Le groupe de randomisation sera inconnu par le médecin examinateur.

***Groupe A:*** traitement par pioglitazone 45 mg/j pendant 16 semaines, puis 2 semaines de wash-out, puis à nouveau par placébo.

***Groupe B :*** placébo 16 semaines, puis 2 semaines de wash-out, puis 16 semaines par pioglitazone 45 mg/j.

Le groupe A sera composé de 6 patients traités par HD et 3 patients traités par DP.

Le groupe B composé de 6 patients traités par HD et 3 patients traités par DP.

Soit un total de 12 patients en hémodialyse et 6 patients en dialyse péritonéale.

Ce design permet de doubler la puissance statistique, puisque chaque patient recevra en fait les 2 traitements et sera son propre contrôle.

## End-points.

**1. Pression artérielle (TA):** *à la fin de chaque phase= 2 x*

**TA pré-dialyse**, TA par **enregistrement ambulatoire de la pression artérielle** (ABPM) de 48h interdialytique

**2. Composition corporelle** par

- **DEXA** (corps entier) : masse musculaire, masse adipeuse

A *la fin de chaque phase = 2 x*

- **une coupe abdominale par CT scan** (tissus viscéral intra-abdominal/tissus sous-cutané).

*A la fin de chaque phase = 2 x*

La quantité de tissu adipeux sera déterminée par une méthode d’absorptiométrie par rayons X (DEXA) avec un appareil Hologic 4500 (Waltham, Mass). La distribution du tissu adipeux sera déterminée par une coupe abdominale par CT scanner (GE, Milwaukee, Wis) à l’étage L4-L5, utilisant des paramètres « low doses » suffisant pour les mesures à réaliser (tissus viscéral intra-abdominal/tissus sous-cutané). L'acquisition des images se fait en utilisant une haute tension de 120 kV et une charge par coupe de 125 mAs (250 mA - 0.5 s) conduisant à une dose moyenne dans la coupe de 12.5 mGy. La largeur d'irradiation minimale est de 1 cm ce qui conduit à une dose effective de 0.19 mSv.

La quantification de la répartition du tissu adipeux se fera à l’aide d’un programme d’analyse segmentaire (Alice software) (Perceptive Systems, Inc., Boulder, CO)Le tissu sous-cutané sera déterminé en sélectionnant les limites internes et externes du tissu adipeux comme région d’intérêt et en comptant le nombre de pixel correspondant à la surface occupée par ce tissu adipeux. Le tissu adipeux intraabdominal (viscéral) sera déterminé en utilisant des histogrammes spécifiques aux viscères.

- **Spectro-IRM**

*A la fin de chaque phase =2x*

Les lipides intramusculaires sont un marqueur de la résistance à l’insuline. Pour cette raison, une évaluation de ces lipides au niveau du muscle tibial antérieur par spectro-IRM sera réalisée.

La résonance magnétique est utilisée de plus en plus pour investiguer la physiologie de la musculature. L’IRM révèle la morphologie du muscle en détail et permet la détermination du volume et l’orientation des fibres musculaires , alors que la spectro-IRM donne des informations sur la composition chimique du tissu musculaire. La spectro-IRM, basée sur l’analyse de la fréquence de résonance des différents métabolites, permet de détecter la présence et de quantifier la graisse intramyocellulaire[10]. Le signal de chaque métabolite est reconnu par son déplacement chimique dans le spectre des protons et sa concentration est quantifiée en calculant l’aire sous la courbe au pic de fréquence de la résonance correspondante. Si le ratio de l’eau et de la graisse dans le tissu musculaire doit être calculé, un volume plus grand est choisi pour moyenner la concentration de plages graisseuses éventuelles. Au contraire, pour séparer le signal de la graisse extramyocellulaire (EMCL) et intramyocellulaire (IMCL) , il faut supprimer le signal de l’eau et bien choisir la région d’intérêts dans le muscle en évitant le tissu sous-cuntané[11].

La spectro-IRM est une méthode non-invasive qui peut être répétée plusieurs fois. La spectro-IRM a le potentiel de remplacer la biopsie musculaire en pouvant documenter les variations de graisse intramyocellulaire[10-13]. Dans une comparaison de différentes methods à disposition pour évaluer la graisse intra et extramyocellulaire, la spectro-IRM et l’histomorphométrie ont montré une valeur diagnostique semblable et l’analyse chimique a le défaut de pouvoir être contaminée par les adipocytes de l’échantillon[10]. En tout état de cause, la spectro-IRM est la méthode la plus constante et la plus fiable parmi elles.

*Collaboration avec Dr N. Theumann, Médecin-Adjoint, Service de radiologie.*

3. **Evaluation de l’appétit** (sur une échelle computérisée visuelle). Carnet alimentaire

A la fin de chaque phase = 2x

*Collaboration avec Prof Roulet, Unité de Nutrition clinique*

4. **Dosages :** Leptine, adiponectine, résistine, TNF-alpha.

Ghréline

Glycémie, insulinémie (HOMA)

CRP sensible

Cholestérol total, HdL, TG

*A la fin de chaque phase = 2x*

###### Service de Néphrologie, volume total de sang =2 x 20ml

5. **Evaluation nutritionnelle anthropométrique** : poids, circonférence abdominale, pli cutanés, circonférence musculaire brachiale (CMB)

#### Mesure de la sensibilité hépatique et extrahépatique à l’insuline

**Evaluation de la** **dépense énergétique et de la lipolyse**.

Biopsie du tissus adipeux sous-cutané

*A la fin de chaque phase = 2x*

Les sujets auront reçu d’une diététicienne des instructions pour consommer une alimentation isoénergétique équilibrée (hydrates de carbone 50%, lipides 35%, protéines 15%). A leur arrivée au Laboratoire d’Investigations Métaboliques situé à la PMU au 7^ème^ étage, les sujets seront pesés et mesurés, les circonférences de leur taille et de leurs hanches relevées et leur composition corporelle sera évaluée par la mesure de l’épaisseur des plis cutanés[14].

Ils prendront ensuite place dans un lit et 2 perfusions seront mises en place dans une veine antécubitale des bras. Une des perfusions servira à la récolte périodique d’échantillons sanguins. Par l’autre perfusion, de l’insuline, du glucose, du glucose deutéré et du ^2^H_5_ glycérol seront perfusés. Un boîtier translucide ventilé avec environ 40 l d’air ambiant/min sera placé sur la tête des sujets pendant toute la durée du test (5h30) et servira à récolter les gaz expirés pour la mesure des dépenses énergétiques et des oxydations de lipides, de glucose et de protéines (calorimétrie indirecte) [15, 16]. Des sondees de microdialyse seront en outre insérées dans le tissu adipeux sous-cutané périombilical (Cf ci-dessous).

Une perfusion d’insuline sera débutée au temps 0. La dose initiale administrée pendant les 90 premières minutes sera de 0.03 mU/kg/min. Au temps 90 min, la dose administrée sera augmentée à 1 mU7kg/min, et ce jusqu’au temps 180 min. Cette perfusion en 2 paliers permet d’obtenir des conditions métaboliques stables pendant les 30 dernières minutes de chaque palier. Le premier palier correspond à une hyperinsulinémie modérée, qui inhibe partiellement la production endogène (hépatique) de glucose et la lipolyse du tissu adipeux ; le second palier correspond à une hyperinsulinémie marquée (concentrations physiologiques après un gros repas), qui inhibe complètement la production de glucose et la lipolyse et stimule de manière non-maximale l’utilisation musculaire de glucose. L’obtention de données métaboliques à ces 2 paliers d’insuline permet donc d’obtenir des informations optimales sur la sensibilité à l’insuline du foie, du tissu adipeux et du muscle. Tout au long des 180 min que dure la perfusion d’insuline, une mesure de la glycémie sera effectuée toutes les 5 minutes , et une perfusion de glucose 20% sera administrée à doses variable de manière à maintenir une glycémie stable à 5 mmol/l ±0.5.

Tout au long de cette période, une perfusion de 6,6 ^2^H_2_ glucose sera administrée (bolus 2 mg/kg, perfusion continue 20 μg/kg/min) et une solution de ^2^H_5_ glycérol sera administrée de manière continue (bolus 1 μmol/kg, perfusion continue 0.1 μmol/kg/min) ainsi qu’une solution de L-[1-^13^C]leucine (Débit de 0,10 µmol.kg^–1^MM.min^-1^ (MM : masse maigre) ou 0,07µmol.kg^–1^PC.min^-1^  (PC : poids corporel) après une injection d’une dose de charge de 8,4 µmol.kg^–1^MM de L-[1-^13^C]Leucine). Des prises de sang seront effectuées toutes les 30 min entre les temps 0 et 180 min pour la mesure des concentrations plasmatiques de glucose, d’insuline, d’acides gras libres, et de glycérol et de l’enrichissement isotopique du 6,6 ^2^H_2_ glucose, du ^2^H_5_glycérol et du L-[1-^13^C]leucine plasmatique.

La lipolyse systémique sera calculée par analyse de dilution isotopique du glycérol plasmatique sur les échantillons prélevés.

La production endogène de glucose sera calculée par analyse de dilution isotopique.

Deux cathéters de microdialyse seront mis en place dans le tissu adipeux périombilical. Un des cathéters sera perfusé avec une solution de Ringer à un débit de 0.3 μl/min. L’autre sera perfusé avec une solution de Ringer additionnée de 10^-6^ M isoprenaline pour stimuler la lipolyse localement. La lipolyse du tissu adipeux sous-cutané (basale et stimulée par l’isoprenaline) sera évaluée en mesurant les concentrations de glycérol dans le dialysate récolté chaque heure. La réalisation de ces mesures en conditations basales et à chaque palier d’insuline permettra d’évaluer la lipolyse du tissu adipeux sous-cutané à jeun, et sa suppression par des doses croissantes d’insuline, ainsi que la sensibilité aux agents béta-adrénergiques

Une biopsie du tissu adipeux sous-cutané seront effectuées sous anesthésie locale et les fragments prélevés seront congelés dans l’azote liquide, puis stockés pour analyse ultérieure de l’expression de différentes molécules à l’aide de microchips et la mesure de mRNA codant pour la leptine, l’adiponectine, la résistine, le TNF-alpha, l’UCP. Volume total de sang : 40ml x 2

*Collaboration avec Prof. L. Tappy, Département de physiologie.*

## Statistiques.

Les méthodes statistiques seront identiques à celles utilisés dans notre publication précédente[17]. Les différences statistiques entre traitement et placebo seront évaluées par ANOVA suivi d’un test de t. Afin d’examiner les effets spécifiques des glitazones chez une personne les valeurs obtenues avec le placebo seront soustraits à celles obtenues avec la pioglitazone avec un test de significance en utilisant le test de t pour une différence significativement différente de 0 (pas de changement avec le traitement). Chaque sujet sera son propre contrôle.

### **Surveillance**

Les patients traités par HD sont vus de routine trois par semaine par le médecin du centre dialyse. D’éventuels effets secondaires seraient donc détectés rapidement. En cas de rétention hydrosodée cliniquement évidente, il sera prescrit une soustraction volémique (ultrafiltration) plus importante lors de la séance de dialyse. L’ultrafiltration est en effet le traitement de choix de l’œdème aigu de poumon chez le patient dialysé anurique.

Les patient traités par DP gèrent leur traitement de dialyse à domicile et ne sont vus qu’une fois par mois de routine à la consultation. Ils devront se peser tous les jours et pourront nous avertir en cas de prise pondérale. Par sécurité, nous leur téléphonerons une fois par semaine pour les questionner au sujet de leur poids ou de l’apparition des oedèmes. En cas d’œdème ou d’apparition de dyspnée liée à une stase pulmonaire, la prescription de solutions de DP hypertoniques ou de polymères du glucose permettrait de soustraire cet excédent d’eau.

Test hépatiques : une fois par mois

Les patients seront exclus de l’étude en cas de rétention hydro-sodée récurrente, d’élévation significative des tests hépatiques (>2.5 x la norme) ou en cas d’autres effets secondaires attribués au traitement de la pioglitazone.

### **Risques**

Pioglitazone : Deux études cliniques préliminaires ont déjà démontrés que les glitazones peuvent être administrés avec sécurité chez des patients traités par HD et DP[4, 5]. Les études pharmacocinétiques montrent que la dose ne doit pas être ajustée et qu’il n’y a pas d’accumulation de la pioglitazone, de la rosiglitazone ou de leurs métabolites[6-9]. Les effets secondaires les plus sérieux, à savoir l’œdème aigu du poumon et la rétention hydrosodée, ne semblent pas plus fréquents que ceux attendus dans cette population[4, 5].

Imagerie :

DEXA/CT-scan: L’irradiation correspond à environ 1/10 de la dose délivrée lors d'un cliché d'abdomen sans préparation. En utilisant un facteur de risque d'induction de cancer mortel de 5% par Sv chez l'adulte, cette acquisition augmente de risque naturel de mort par cancer (qui est d'environ de 0.3) de 0.00001 (10^-5).

La spectro-IRM est une méthode non-invasive qui peut être répétée plusieurs fois. La mesure des lipides et du glycogène intramyocellulaire et intrahépatocytaire par résonance magnétique nucléaire ne comporte pas de risque si les critères d’exclusion sont respectés. Le personnel travaillant au Centre de résonance magnétique nucléaire obéit à des règles de sécurité strictes pour éviter tout accident causé par des objets ferro-magnétiques. Les volontaires seront informés et contrôlés avant d’entrer dans la pièce où se trouve l’aimant.

Clamp/Calorimétrie indirecte/microdialyse/biopsie sous-cutané.

La réalisation de perfusion d’insuline comporte un risque potentiel d’hypoglycémie, rendu cependant négligeable par le fait que la glycémie est mesurée toutes les 5 minutes au lit du malade et qu’une perfusion de glucose 20% est en place.

La pose de sonde de microdialyse dans le tissu adipeux sous-cutané périombilical s’effectue de manière aseptique sous anesthésie locale à la xylocaïne. Il existe un risque très faible de rupture de la sonde de microdialyse (1 cas à Lausanne sur plus de 300 sondes posées au cours des 5 dernières années). Un tel incident, s’il survenait, ne représente pas de risque majeur, le matériel dont les sondes sont constituées étant biocompatible. Il est toutefois possible que le développement ultérieur d’un granulome nécessite une extirpation chirurgicale locale.

Les biopsies du tissu adipeux sous-cutané comportent un risque d’hématome et d’infection locale. Ce dernier est cependant minimisé par l’emploi de techniques d’asepsie stricte.

**Désagréments de l’étude pour les patients :** Il s’agit de 4 demi-journées d’investigations en dehors des jours habituels de dialyse, ceci sur une période de plus de 8 mois. Lors de 2 demi-journées, deux cathéters seront posés dans les avants-bras. Les patients devront prendre des pilules supplémentaires chaque jour (un pilule de plus, placebo ou pioglitazone) pendant une période de 8 mois. En dehors des 4 demi-journées d’investigations, les patients hémo-dialysés seront vus comme d’habitude, soit trois fois par semaine lors des scéances de dialyse. Les patients dialysés péritonéaux devront en revanche être vus de façon hebdomadaire au lieu de mensuelle pendant la période de cette étude.

**Personnel infirmier :** Le personnel infirmier impliqué fera son travail de routine chez les dialysés ou dans le cadre du Laboratoire d’Investigations Métaboliques ou du CIBM.

**Médicaments :**

- pioglitazone 45mg (Actos**®**), gélules préparés à partir du lots disponibles sur le marché suisse pour le traitement des patients.

La dose choisie de 45mg de pioglitazone correspond à la dose maximale. L’effet métabolique de la pioglitazone atteint son pic dans les 6-12 semaines après le début du traitement. Compte tenu de la durée du traitement de seulement 12 semaines, nous avons choisi la dose de 45mg.

## - insuline : Actrapid HM® 100 U/ml, Novo Nordisk

- glucose : glucose 20%, B Braun

- 6,6 ^2^H_2_ glucose : solution injectable10 g/l

^- 2^H_5_ glycérol : solution injectable 10 g/l

- Solution de Ringer + isoprenaline

Pharmacie centrale, CHUV

**Aspects éthiques**

La volontaire recevra des informations détaillées, par oral du médecin, et par écrit dans le formulaire d’information. Le consentement éclairé écrit de chaque sujet sera obtenu, après un délai de réflexion.

A tout moment, les volontaires pourront se retirer de l’étude sans donner de motifs, ils en informeront cependant un des responsables de l’étude. Toutes les données récoltées seront confidentialisées. Chaque volontaire sera identifié par un numéro.

**Responsabilité civile de l’investigateur principal : assurance RC professionnelle du CHUV**

.

Les patients/volontaires participant à cette étude seront couverts par l’assurance RC du CHUV. En tant que sponsor de l’étude, le service de Néphrologie sera couvert par l’Etat de Vaud, le CHUV n’ayant plus d’assurance couvrant les médecins de l’Institution en tant que sponsor. En cas de complication survenant au cours de l’étude, les sujets auront droit à une compensation pleine et entière

**Sources de financement et rétribution:**

Cette étude est conduite par la Service de Néphrologie qui est le sponsor de l’étude. Pour leur participation, les sujets recevront un dédommagement de 800 CHF. Les examens de laboratoire seront pris en charge par le service et les examens radiologiques seront assumés par le Service de Radiologie dans le cadre d’une collaboration scientifique. Aucun montant ne sera à la charge du sujet de recherche.

#### Références

1. Chiquette E, Ramirez G, Defronzo R: A meta-analysis comparing the effect of thiazolidinediones on cardiovascular risk factors. *Arch Intern Med* 164:2097-2104, 2004

2. Smith SR, De Jonge L, Volaufova J, Li Y*, et al.*: Effect of pioglitazone on body composition and energy expenditure: a randomized controlled trial. *Metabolism* 54:24-32, 2005

3. Miyazaki Y, Mahankali A, Matsuda M, Mahankali S*, et al.*: Effect of pioglitazone on abdominal fat distribution and insulin sensitivity in type 2 diabetic patients. *J Clin Endocrinol Metab* 87:2784-2791, 2002

4. Manley HJ, Allcock NM: Thiazolidinedione safety and efficacy in ambulatory patients receiving hemodialysis. *Pharmacotherapy* 23:861-865, 2003

5. Lin SH, Lin YF, Kuo SW, Hsu YJ*, et al.*: Rosiglitazone improves glucose metabolism in nondiabetic uremic patients on CAPD. *Am J Kidney Dis* 42:774-780, 2003

6. Budde K, Neumayer HH, Fritsche L, Sulowicz W*, et al.*: The pharmacokinetics of pioglitazone in patients with impaired renal function. *Br J Clin Pharmacol* 55:368-374, 2003

7. Chapelsky MC, Thompson-Culkin K, Miller AK, Sack M*, et al.*: Pharmacokinetics of rosiglitazone in patients with varying degrees of renal insufficiency. *J Clin Pharmacol* 43:252-259, 2003

8. Thompson-Culkin K, Zussman B, Miller AK, Freed MI: Pharmacokinetics of rosiglitazone in patients with end-stage renal disease. *J Int Med Res* 30:391-399, 2002

9. Snyder RW, Berns JS: Use of insulin and oral hypoglycemic medications in patients with diabetes mellitus and advanced kidney disease. *Semin Dial* 17:365-370, 2004

10. Howald H, Boesch C, Kreis R, Matter S*, et al.*: Content of intramyocellular lipids derived by electron microscopy, biochemical assays, and (1)H-MR spectroscopy. *J Appl Physiol* 92:2264-2272, 2002

11. Boesch C, Decombaz J, Slotboom J, Kreis R: Observation of intramyocellular lipids by means of 1H magnetic resonance spectroscopy. *Proc Nutr Soc* 58:841-850, 1999

12. Boesch C, Kreis R: MR-spectroscopy (MRS) of different nuclei applied to human muscle: additional information obtained by 1H-MRS. *Int J Sports Med* 18 Suppl 4:S310-312, 1997

13. Boesch C, Kreis R: Observation of intramyocellular lipids by 1H-magnetic resonance spectroscopy. *Ann N Y Acad Sci* 904:25-31, 2000

14. Durnin JV, Rahaman MM: The assessment of the amount of fat in the human body from measurements of skinfold thickness. *Br J Nutr* 21:681-689, 1967

15. Dirlewanger M, di Vetta V, Guenat E, Battilana P*, et al.*: Effects of short-term carbohydrate or fat overfeeding on energy expenditure and plasma leptin concentrations in healthy female subjects. *Int J Obes Relat Metab Disord* 24:1413-1418, 2000

16. Schneiter P, Tappy L: Kinetics of dexamethasone-induced alterations of glucose metabolism in healthy humans. *Am J Physiol* 275:E806-813, 1998

17. Zanchi A, Chiolero A, Maillard M, Nussberger J*, et al.*: Effects of the peroxisomal proliferator-activated receptor-gamma agonist pioglitazone on renal and hormonal responses to salt in healthy men. *J Clin Endocrinol Metab* 89:1140-1145, 2004
