# Supplementary material for: Pioglitazone Improves Fat Distribution, the Adipokine Profile and Hepatic Insulin Sensitivity in Non-Diabetic End-Stage Renal Disease Subjects on Maintenance Dialysis: A Randomized Cross-Over Pilot Study
Source: PLoS One. 2014 Oct 16;9(10):e109134. doi: 10.1371/journal.pone.0109134 (PMC4199598; doi:10.1371/journal.pone.0109134)
Supplement: Protocol S2 — Trial Protocol (translation). (DOCX) [file pone.0109134.s003.docx]

| Dr Daniel TETA, Médecin adjoint, PD & MER  Tél : 021 314 11 54 (secr)  Tél : 021 314 11 31 (desk)  Fax : 021 314 11 39  daniel.teta@chuv.ch |  |
| --- | --- |

Lausanne, 11th January 2007

**Effect of glitazones on insulin resistance and body composition in patients with end stage renal disease**

**Investigators :** Dr PD A Zanchi, MD, Service de Néphrologie

Dr D Teta MD-PhD, Service de Néphrologie

Prof L Tappy, MD, Département de physiologie

Dr PD-MER N Theumann, MD, Service de radiologie

Dr G Halabi, MD, Service de Néphrologie

Dr T Gauthier MD, Service de Néphrologie

Dr M Maillard, PhD, Service de Néphrologie

Prof M Roulet, MD, Unité de Nutrition clinique

Prof. Michel Burnier, MD, Service de Néphrologie

**Introduction :**

Patients with end stage renal disease (ESRD) on dialysis have high cardiovascular mortality, accelerated atherosclerosis, hypertension, accumulation of central fat, a high prevalence of protein energy wasting and inflammation. These abnormalities are all associated with insulin resistance (IR) which appears to be central in the pathogenesis of these complications.

Glitazones are oral antidiabetic drugs known to reduce IR. These drugs act as agonists of peroxysome proliferator agonists gamma (PPAR-g), which are abundantly expressed in the adipose tissue. Glitazones are essentially prescribed in type 2 diabetes patients to reduce glycemia but they also have pleiotropic effects. In particular, glitazones reduce blood pressure and have anti-atherogenic and anti-inflammatory actions, which make them potentially valuable for dialysis patients.

Glitazones are known to induce adipose tissue redistribution, from visceral (central) to subcutaneous adipose tissue. They also stimulate the differentiation of pre-adipocytes into « more favorable adipocytes », increase plasma adiponectin (an insulin-sensitizing adipokine), reduce inflammation and this contribute to improve dyslipidemia and blood pressure control. Overall glitazones induce a mean weigh gain of 3 kg (1). Some studies showed that there is accumulation of subcutaneous adipose tissue, together with a reduction of visceral adipose tissue (2,3). Glitazones have no effect on energy expenditure. This is why the increase in body weight may be due to an increase in calorie intake, estimated to 175 kcal/day in the latter study, although food questionnaires failed to show differences between glitazones and placebo users. In an animal model of IR, where calorie intake is readily measurable, we were able to show that glitazones clearly increase calorie intake. In addition to ESRD, the dialysis technique itself has a specific impact on body composition. In particular patients on peritoneal dialysis (PD) tend to accumulate more adipose tissue than patients treated by hemodialysis (HD). As a consequence, we would like to investigate whether the effect of glitazones on body composition may differ between treatment modalities.

Two preliminary studies have shown that glitazones may be safely prescribed both in HD and PD patients (4,5). Pharmacokinetic studies showed no accumulation of pioglitazone and rosiglitazone and their metabolites, and thus their dosage should not be adjusted to renal function (6,7,8,9). The more serious side effects, i.e. pulmonary edema and fluid overload do not appear more prevalent than expected in this population (4,5).

**Aim:**

The aim of this study is to investigate the effect of glitazones on IR, body composition, plasma adipokines and inflammation in patients on HD and PD.

We expect that this treatment, induce fat redistribution towards subcutaneous adipose tissue, reduce inflammation, improve plasma adipokine profile, while reducing IR. If well tolerated, this treatment would in this case reduce cardiovascular is at long term in this population.

**Study design:**

Single centre, randomized, placebo-controlled trial, glitazone versus placebo. Each phase lasts 16 weeks.

Inclusion criteria

1. Patients treated with HD or PD since at least 3 months recruited at Lausanne University Hospital.
2. Patients not hospitalized, and with no active infection in the last 3 months before inclusion.
3. CRP < 20 mg/L.
4. Consent form signed.

Exclusion criteria

1. Any current inclusion in another protocol.
2. Use of glitazone or insulin.
3. Estimated survival less than 6 months.
4. Heart failure class III and IV, according to NYHA.
5. ASAT, ALAT increased levels of > 2.5 fold and/or active liver disease.
6. Women without contraception.
7. Positive pregnancy test.

**Protocol**

Patients will be randomized into 2 groups, blinded to the patients and the physicians.

Group A: Treatment with pioglitazone 45 mg/day for 16 weeks, than 2 weeks of wash-out, than placebo 16 weeks.

Group B: Placebo 16 weeks, than wash-out 2 weeks, than pioglitazone 45 mg/day for 16 weeks.

**Design of the study**

## A graph summarizing the design is show below:

##

## End-points

1) Body composition (2x = at the end of each phase):

- Total and regional body composition by Dual-Energy X-ray Absorptiometry (DXA)
- Visceral and sub-cutaneous fat areas by computed tomography (level L4-L5)
- Anthropometric measurements: Weight, abdominal circumference, skin folds

2) Plasma adipokines (2x = at the end of each phase):

- Leptin, adiponectin, TNF-a

3) Inflammation parameters (2x = at the end of each phase):

- TNF-a, CRP

4) Blood lipids (2x = at the end of each phase):

- Total cholesterol, HDL-cholesterol, triglycerides

5) Energy expenditure study (2x = at the end of each phase):

- Indirect calorimetry

6) Glycemia and insulin sensitivity (2x = at the end of each phase):

- HbA1C, glucose, insulin sensitivity (HOMA index)
- Insulin-glucose clamp (2x)

**Follow-up:**

HD patients are routinely seen 3x a week at the dialysis centre. Thus, fluid retention will be detected easily. PD patients will be seen 1x/week and fluid retention will be carefully searched for. In case of fluid retention, the dialysis treatment will be adapted to promote more fluid extraction.

Liver function tests: 1x/month

### **Risks**

Pioglitazone: Two studies have already shown that glitazones can be administered safely in HD and DP patients [4, 5]. Pharmacokinetic studies have shown that the dose should not be adjusted according to renal function since there is no accumulation of pioglitazone, rosiglitazone or their metabolites [6-9]. Serious adverse events such as pulmonary edema and fluid overload do not seem to be more frequent than expected in this population [4, 5].

Imaging studies: DXA/CT-scan:

The exposure to radiation is equivalent to 1/10 of the dose delivered for a plain abdomen Xray.

Insulin-glucose clamp/ Indirect calorimetry

Insulin infusion may be associated with a risk of hypoglycemia, which is in fat negligible since glycemia will be measured every 5 minutes at bed site and that a 20% glucose infusion is perfused simultaneously.

Inconveniences for the patients:

Over a period of 8 months, there will be 4 sessions (half-a-day) of investigations during the days not dedicated to hemodialysis sessions (for those who are treated with HD). For PD patients, treatment is continuous and will be just interrupted during the investigation (no PD fluid in the abdomen during the investigation).

Out of these 4 sessions, 2 sessions will be dedicated to the study of hyperinsulinemic euglycemic clamps. During these 2 sessions, 2 intravenous catheters will be placed in the forearms.

During the whole study, i.e. 8 months, patients will be asked to take 1 supplementary pill per day (placebo or pioglitazone).

**Drugs used in this study:**

Pioglitazone 45mg (Actos**®**)

Pills prepared from package already available in the swiss market. The dose of 45mg de pioglitazone is equivalent to the maximal dose. The metabolic effect of pioglitazone reaches its peak at 6-12 weeks after the start of the treatment. Given the relatively short treatment duration, we have decided to choose the dose of 45 mg.

## The following drugs will be obtained at the central pharmacy at the University Hospital of Lausanne:

## insuline : Actrapid HM® 100 U/ml, Novo Nordisk

- glucose : glucose 20%, B Braun
- 6,6 ^2^H_2_ glucose : solution injectable10 g/l
- ^2^H_5_ glycérol : solution injectable 10 g/l
- Ringer solution + isoprenaline

**Ethical aspects :**

The volunteer will receive detailed information, orally by the physician in charge (nephrologist), and through the patient’s information form. A written consent will be obtained.

Volunteers have the possibility to withdraw from the study, without necessarily giving their reasons. In this case, they will have to inform one of the investigators of the study. All data collected will be confidentially treated. Volunteers will be given a study number.

**Insurances and responsability of the investigators:**

Volunteers are covered by the hospital insurance. As a sponsor of the study, the Service of Nephrology is covered by the Canton de Vaud. In the case of damaged in relation with the study, the subjects will be reimbursed according to the damage.

**Financial issues:**

This study is conducted by the Service de Nephrology, which is the sponsor of the study. Subjects will receive 800.-CHF for the time and inconveniences related with the study. Laboratory analysis will be covered by the Service of Nephrology. Radiologic imaging will be covered by the Department of Radiology which has been integrated in the scientific collaboration. Volunteers and /or their private insurance will not be charged.

**Impact of the study:**

Glitazone treatment is expected to improve insulin resistance, decrease blood pressure and inflammatory markers as well as increase body weight while decreasing visceral fat and improving the nutrition status. This study will provide in detail the metabolic modifications induced by glitazone treatment in ESRD patients treated by HD and PD.

## Perspectives:

## Insulin resistance characterises patients with end-stage renal disease. Prior to the availability of glitazones, no insulin sensitising drugs could be prescribed to ESRD patients. Glitazones may improve the poor nutritional and cardiovascular morbidity of ESRD and chronic renal failure patients.

**References**

1. Chiquette E, Ramirez G, Defronzo R: A meta-analysis comparing the effect of thiazolidinediones on cardiovascular risk factors. *Arch Intern Med* 164:2097-2104, 2004

2. Smith SR, De Jonge L, Volaufova J, Li Y*, et al.*: Effect of pioglitazone on body composition and energy expenditure: a randomized controlled trial. *Metabolism* 54:24-32, 2005

3. Miyazaki Y, Mahankali A, Matsuda M, Mahankali S*, et al.*: Effect of pioglitazone on abdominal fat distribution and insulin sensitivity in type 2 diabetic patients. *J Clin Endocrinol Metab* 87:2784-2791, 2002

4. Manley HJ, Allcock NM: Thiazolidinedione safety and efficacy in ambulatory patients receiving hemodialysis. *Pharmacotherapy* 23:861-865, 2003

5. Lin SH, Lin YF, Kuo SW, Hsu YJ*, et al.*: Rosiglitazone improves glucose metabolism in nondiabetic uremic patients on CAPD. *Am J Kidney Dis* 42:774-780, 2003

6. Budde K, Neumayer HH, Fritsche L, Sulowicz W*, et al.*: The pharmacokinetics of pioglitazone in patients with impaired renal function. *Br J Clin Pharmacol* 55:368-374, 2003

7. Chapelsky MC, Thompson-Culkin K, Miller AK, Sack M*, et al.*: Pharmacokinetics of rosiglitazone in patients with varying degrees of renal insufficiency. *J Clin Pharmacol* 43:252-259, 2003

8. Thompson-Culkin K, Zussman B, Miller AK, Freed MI: Pharmacokinetics of rosiglitazone in patients with end-stage renal disease. *J Int Med Res* 30:391-399, 2002

9. Snyder RW, Berns JS: Use of insulin and oral hypoglycemic medications in patients with diabetes mellitus and advanced kidney disease. *Semin Dial* 17:365-370, 2004
